# Efficacy of In Vitro Lithium Chloride Treatments on *Dermacentor reticulatus*

**DOI:** 10.3390/insects14020110

**Published:** 2023-01-20

**Authors:** Balázs Kolics, Kinga Mátyás, Izabella Solti, Zsuzsanna Bacsi, Szilvia Kovács, András Specziár, János Taller, Éva Kolics

**Affiliations:** 1 Festetics Bioinnovation Group, Department of Microbiology and Applied Biotechnology, Institute of Genetics and Biotechnology, Georgikon Campus, Hungarian University of Agriculture and Life Sciences, H-8360 Keszthely, Hungary; 2 Department of Agricultural Economics and Policy, Institute of Agricultural and Food Economics, Georgikon Campus, Hungarian University of Agriculture and Life Sciences, H-8360 Keszthely, Hungary; 3 Department of Wildlife Biology and Management, Institute for Wildlife Management and Nature Conservation, Georgikon Campus, Hungarian University of Agriculture and Life Sciences, H-8360 Keszthely, Hungary; 4 Balaton Limnological Research Institute, H-8237 Tihany, Hungary

**Keywords:** lithium chloride, lithium, biocidal effect, dog tick, acaricide

## Abstract

**Simple Summary:**

*Dermacentor reticulatus* is a significant parasite and vector of various pathogens which are dangerous to humans and animals. Lithium chloride is currently one of the most promising potential alternatives against *Varroa destructor*, a detrimental mite parasite of honeybees. Furthermore, it shows efficacy against *Dermanyssus gallinae*, a severe pest of poultry, in vitro. In the present study, we report for the first time that the biocidal efficacy of lithium chloride extends to *D. reticulatus* as well, with 100% mortality at a minimum concentration of 1.38 M and an LC_50_ of 0.654 M. Our results may contribute to a comprehensive understanding of the lithium ion.

**Abstract:**

*Dermacentor reticulatus* (Fabr., 1794) (Acari: Ixodidae) is parasite that spreads many diseases which are dangerous to humans and animals. Microelement lithium was found to have promising potential against the detrimental bee pest *Varroa destructor.* Furthermore, its effectiveness was confirmed against *Dermanyssus gallinae,* a major parasite of poultry, in vitro. In the present study, we investigated whether the efficacy of lithium chloride extends to other parasitic species, such as *D. reticulatus*. Our results revealed, for the first time, that the effectiveness of lithium chloride extends to *D. reticulatus*, confirmed to have 100% mortality at a relatively high minimum concentration of 1.38 M in vitro. The 24 h and 48 h median lethal concentration (LC_50_) values proved to be 0.654 M and 0.481 M, respectively, for this species. Our pilot study may contribute to a better understanding of the properties of lithium ion. Furthermore, it may elicit further studies aiming to reveal whether the different environmental mineral conditions may influence the *D. reticulatus* population. Further studies might reveal whether lithium has any possible veterinary relevance.

## 1. Introduction

Ticks belong to the arthropod class Arachnida and superorder Parasitiformes. These transient parasites mediate several diseases, causing local skin lesions and inflammatory reactions. Controlling ticks is important and necessary as they are vectors for many human and animal pathogens. Attempts to control ticks include destroying their habitats or using repellent agents and pesticides. The most commonly used active substances for tick control are imidacloprid, fipronil, and permethrin and their combinations [1,2]; (S)-methoprene; pyriprole; metaflumizone; and amitraz and deltamethrin [3]. However, control efforts are limited due to resistance, and acaricides can cause environmental pollution and pose a risk of residues. Acaricides based on natural products, such as biological substances derived from plants [4], may be an alternative. Another potential control method is the use of antibiotics, as treatment can disrupt the homeostasis of the tick microbiome, reducing tick fitness and affecting tick–pathogen interactions. Ticks live and interact with pathogenic microorganisms, symbionts, and commensal bacteria, forming an ecological unit called the tick holobiont, which is also subject to natural selection. In a novel study, Wu-Chuang and colleagues hypothesized that targeting key members of the bacterial community of the tick microbiome with antibodies could cause microbial dysbiosis [5]. Gu and colleagues tested the effects of lithium chloride solution on four airborne pathogenic microbes (*Bacillus subtilis*, *Staphylococcus aureus*, *Pseudomonas aeruginosa,* and *Aspergillus niger*). The results showed that the lithium chloride solution was remarkably effective, inactivating pathogens by 35.2–96.2% within 60 min [6].

The efficacy of lithium chloride also extends to parasitic mites, such as *Varroa destructor* (*Arachnida*: *Acari*: *Varroidae*). This mite is a vector of several bee viruses and represents a major threat to apiculture, as it can kill a whole colony of bees in a single season. Ziegelmann et al. reported that lithium chloride effectively eradicated *V. destructor* in in vitro feeding experiments [7]. Later, it was confirmed that its strong biocidal effect contributes to the high efficacy of the varroacide agent [8], and techniques relevant to beekeeping practice have been published for lithium-based *Varroa* control treatments [9]. Additionally, it has been recently revealed that lithium is effective against the poultry red mite in vitro [10].

Due to taxonomic proximity and the antibacterial effect of lithium, which may also affect tick holobiont, in this study, we investigated the efficacy of lithium chloride on the dog tick (*D. reticulatus*). This hard tick species is found in meadows, open woodlands, heaths, glades, suburban grasslands, and marshes, and prefers habitats with high humidity [11,12]. Fertilized females can lay up to 7200 eggs [13], and adults are highly tolerant to changing environments. Adults are exophilic and infest larger mammals, such as dogs, horses, goats, sheep, cattle [14], and occasionally humans [15]. This species is a vector of more than 40 different veterinary and medically important pathogens [11]. Dog ticks are the most important mediator of several babesiosis diseases (*Babesiosis divergens*, *Babesiosis microti*, *Babesiosis odocoilei*, *Babesiosis canis*, and *Babesiosis caballi*) [16,17,18,19,20,21]. In western and central Europe, a northward spread of babesiosis has been observed in recent years, likely due to climatic changes that increase ticks’ survival and cause host animals to travel [22,23]. It is also a vector of several bacterial diseases, such as granulocytic ehrlichiosis, Lyme borreliosis, tick-borne lymphadenopathy (TIBOLA), and tularemia [20,24,25,26,27]. It has recently been shown that *D. reticulatus,* like *Ixodes ricinus*, can also transmit tick-borne encephalitis virus (TBEV) [26,28].

Since *D. reticulatus* is a vector of many severe diseases and its range has been increasing recently, we aimed to investigate whether the efficacy of lithium may extend to this tick species under in vitro conditions.

## 2. Materials and Methods

### 2.1. Sampling

A total of 790 individuals of *D. reticulatus* were used in treatments (Table 1). The adult dog ticks were of mixed age and sex, collected in July 2021 from Balatonszentgyörgy (N: 46.6902°, E: 17.2973°) and Keszthely (N: 46.766°, E: 17.257°), Hungary. Samples were collected from three locations in both areas, separated by at least 1000 m, and mixed. At least 15 *D. reticulatus* individuals were used to test each concentration.

### 2.2. Immersion Contact Test

Two separate tests were conducted to describe the dynamics of response and to determine the LC_50_ values for the lithium chloride in dog ticks. In Test I, aqueous lithium chloride solutions (deionized water, LiCl a.r, Szkarabeusz Kft.,Pécs, Hungary) were used in the following concentrations: 5.520 M, 2.760 M, and 1.380 M, similar to those applied in previous studies on *V. destructor* and *D. gallinae* [8,10]. The dog ticks were immersed in 1 mL solutions in Eppendorf tubes (Thermo Fisher Scientific, Waltham, MA, USA) and slightly vortexed for 10 s to remove any air bubbles on the animals, ensuring an even contact exposure. Subsequently, they were placed on a filter disc (Sartorius, d = 150 mm, Grade: 1292; Thermo Fisher Scientific, Waltham, MA, USA) and placed in Petri dishes. The first recorded event was the onset of tremorous, uncontrollable movements. The second event was recorded when the dog tick lost locomotion, but responded to stimuli. Subsequently, the time of death was recorded as the third event. The control treatment was carried out with ion-exchanged water only (*n* = 50).

Test II was conducted to determine the concentration (LC_50_) at which lithium chloride would kill half of the tested dog ticks. To establish a concentration–response relationship, the mortality of dog ticks was tested at 15 concentrations ranging between 5.520 M and 0.000 M (control), as specified in Table 1. The treatment procedure was identical to that applied in Test I. The only monitored symptom was death at 24 h and 48 h post-treatment.

In Test III, the mortality of dog ticks was evaluated at six concentrations of aqueous sodium chloride solutions ranging from 5.000 M to control, 0.000 M (Table 1), following an immersion procedure identical to that applied in Test I and Test II. This experiment aimed to determine whether lithium, rather than the chloride ion or high salt concentration, was responsible for the effectiveness of lithium chloride against ticks.

The experiments were conducted at 22 °C, with 50% relative humidity maintained using humidity solutions, under a 12-hour photoperiod.

### 2.3. Statistical Analysis

For statistical analysis in Test I, Abbott’s formula [29] was used to calculate the mortality rates for 147 observations of *D. reticulatus*. The sample sizes varied, with 31 animals observed for concentrations of 1.380 M, 19 for 2.760 M, 47 for 5.520 M, and 50 for the control. Extreme values were identified, and 6 cases (5 for concentration 1.380 M and 1 for concentration 5.520 M) were excluded from further analysis because they exceeded 3 times the interquartile range. The data were transformed using the natural logarithm (ln transformation) and tested for normality using the Jarque–Bera and Shapiro–Wilk tests. The *ln*-transformed data for each mortality stage were found to be normally distributed (*p* > 0.05). One-way ANOVA was used to test for significant differences between the natural logarithms of exposure times to each stage (uncontrolled movement, inability to move, and death). The Levene test was used to justify homogeneous variances, while the Welch and Brown–Forsythe tests were applied when the assumption of homogeneity of variances was violated. These statistical tests were computed by SPSS 22.0 software (IBM, New York, NY, USA).

In Test II, the Hill model (i.e., 4-parameters logistical) [30] was used to fit the mortality data of dog ticks exposed to lithium chloride concentrations, and the LC_50_ value was calculated using the Quest Graph™ LC_50_ Calculator from AAT Bioquest Inc. (Sunnyvale, CA, USA) [31].

No statistical testing was required in Test III due to the absence of differences. 

## 3. Results

**Figure 1:**
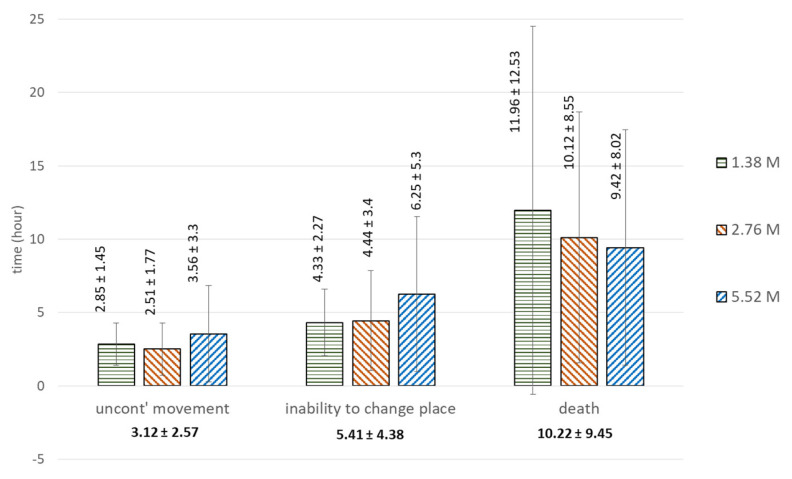
Mean time ± SD (hours) from immersion to the mortality stages in Test I, by lithium chloride concentration (note: treatment concentrations had no significant effect on the exposure time to reach any of the mortality stages).

In Test I, all of the treated groups (i.e., those exposed to 5.52 M, 2.76 M, and 1.38 M lithium chloride) exhibited symptoms of poisoning, while the control group did not show any symptoms of poisoning (i.e., uncontrolled movement, inability to move, or death). Although the average exposure times to reach the stages of uncontrolled movement, inability to move, and death showed some variation between concentrations, these differences were statistically insignificant (Figure 1 and Table 2). Specifically, the average exposure time to reach lethal time (LT_100)_ (i.e., death) appeared to decrease with increasing concentrations, but the sizable variation in the data, as indicated by the standard deviations (SD), made the differences statistically insignificant (one-way ANOVA, *p* = 0.798).

**Figure 2:**
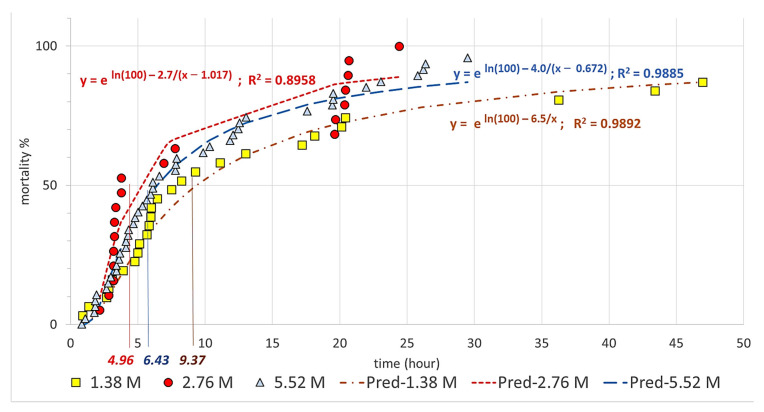
Abbott-corrected mortality rates for the three lithium chloride concentrations.

As is shown in Figure 2, a Johnson-type logistic growth curve was fitted to the Abbott corrected mortality rate data for each concentration. The curve had the form y = e ^K−b/(x−c)^ where e^K^ = 100 (i.e., *K* = 4.61), *y* represents the mortality rate, and *x* represents the exposure time (hours). The parameters *c* and *b* defined the position and slope of the fast growth section of the curve, with larger b values resulting in a steeper curve and smaller c values leading to a later start of the steep increase. The fitted equations are shown in Figure 2 and in Table 2, along with the respective R^2^ values (all greater than 0.89). The figure and the table indicate that as the concentration increased from 1.38 M to 2.76 M or 5.52 M, the speed of mortality increased, while there was little difference between concentrations of 2.76 M and 5.52 M in this respect.

Table 2 shows the average exposure times required to reach the stages of uncontrolled movement, inability to change position, and death at three lithium chloride concentrations.

As the experiments show, a concentration of 2.76 M resulted in the shortest average time required for half of the treated ticks to die, while a concentration of 5.52 M resulted in the shortest average time required to kill the entire treated population.

**Figure 3:**
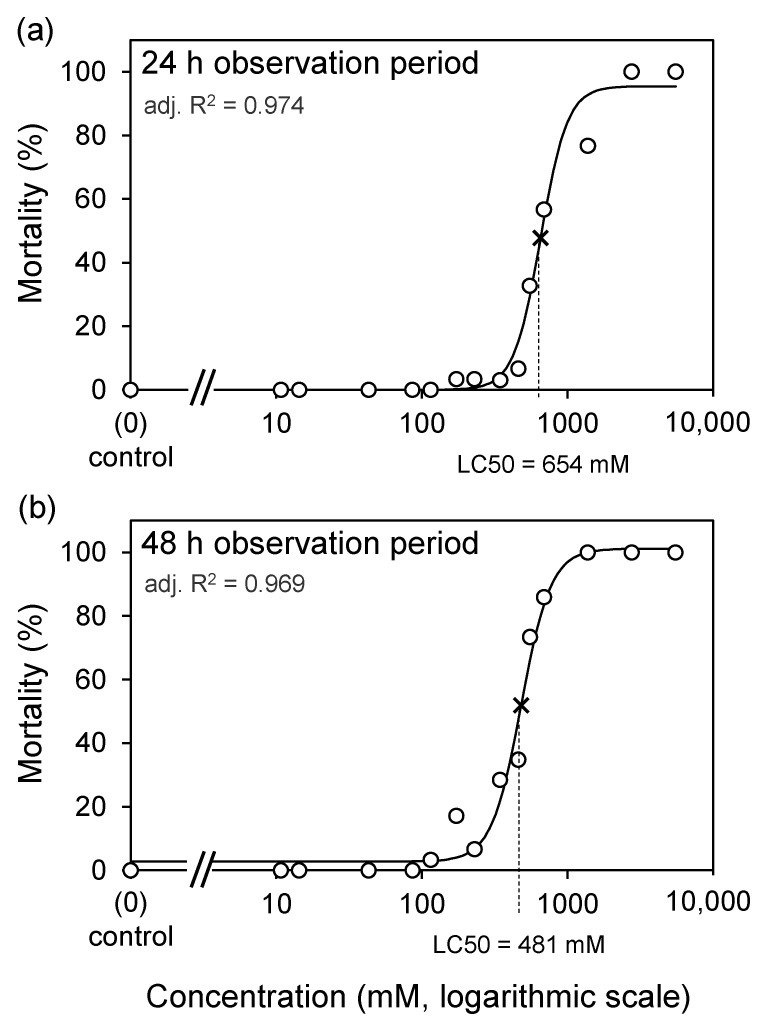
Concentration–response curves and LC_50_ values for lithium chloride in the dog tick for 24 h (**a**) and 48 h (**b**) observation periods.

Test II revealed that the 24 h and 48 h LC_50_ values for lithium chloride in the dog tick were 0.654 M (95% CI: 0.624–0.684) and 0.481 M (95% CI: 0.458–0.503), respectively (Figure 3).

In Test III, none of the sodium chloride-treated individuals or controls showed any signs of poisoning, and all remained alive and symptom-free for the entire test period (24 h).

## 4. Discussion

Our results demonstrate that lithium chloride is effective against the dog tick in a contact mode of action under in vitro conditions. The three highest concentrations (i.e., 1.38, 2.76, and 5.52 M) showed 100% efficacy. The symptoms of lithium poisoning were similar to those of *Varroa* mites. Previous studies suggest that lithium chloride may be of practical importance as an acaricide in beekeeping [7,8,32,33,34], with methods that could be easily integrated into pest management in apiculture if further research on residues in honey supports its use [9].

The use of certain lithium salts as a varroacide raises the possibility of a less environmentally damaging and residue-free future agent, as many foods naturally contain trace amounts of lithium, and previous research on residues in beekeeping has not been regarded as alarming [32,35]. In addition to being the 27th most abundant element in the earth’s crust [36] and a natural component of mineral waters and foods [37,38,39,40], lithium is a trace mineral with a proposed recommended daily intake of 1.0 mg lithium/day for adults [38]. It is also used in human medicine to treat bipolar disorder [41], although it is often associated with side effects at several times the range of trace amounts (~170 mg Li+/day) [42].

Present results, for the first time, suggest that lithium may be effective against dog ticks, with an LC_50_ effect at a concentration of 654 mM after one day. It is important to note that the immersion tests ensured even exposure, providing full contact for each individual, which is unlikely to happen in animals treated with lithium chloride solutions or if these solutions are used to control questing ticks.

These data might encourage further research on lithium as a potential acaricide. Additionally, studies are needed on the effects of lithium on the holobiont of certain tick species, raising the possibility that lithium, as a secondary pesticide, may contribute to tick population reduction by altering the microbiome.

Since parasite eradication can be achieved either by treating the area or the animals, further studies and impact assessments are needed to evaluate the veterinary relevance of lithium. However, the main areas of interest are to understand whether there is a correlation between the lithium content of a given area and the density of the tick population, whether there are differences in the sensitivity of different tick species, and what physiological explanation this may have.

## Figures and Tables

**Table 1:** The number of dog ticks tested (**N**) for the effect of lithium chloride (LiCl) in Tests I and II, and for the effect of sodium chloride (NaCl) in Test III.

Concentration LiCl (Test I & II)	Test I N	Test II N	Concentration NaCl (Test III)	Test III N
5.520 M	47	30	5.000 M	30
2.760 M	19	30	2.500 M	30
1.380 M	31	30	1.250 M	30
0.690 M		30	0.625 M	30
0.552 M		31	0.316 M	30
0.460 M		27		
0.345 M		30		
0.230 M		30		
0.173 M		29		
0.115 M		30		
0.086 M		59		
0.043 M		29		
0.014 M		30		
0.011 M		15		
0.000 (control)	50	37		30
Total	147	463		180

**Table 2:** Mean ± SD for exposure times (hours) by lithium chloride concentrations in Test I.

Concentration	Onset of Uncontrolled Movement	Inability to Change Place	Death
1.38 M	2.85 ± 1.45	4.33 ± 2.27	11.96 ± 12.53
2.76 M	2.51 ± 1.77	4.44 ± 3.4	10.12 ± 8.55
5.52 M	3.56 ± 3.3	6.25 ± 5.3	9.42 ± 8.02
ANOVA		F = 0.603, *p* = 0.549	F = 0.266, *p* = 0.798
Welch Test	F = 0.910, *p* = 0.409		
Brown–Forsythe Test	F = 0.743, *p* = 0.479		
	Trend of mortality rates (y) by exposure times (x)		Estimated time to LT_50_
1.38 M	y = e^ln(100)−6.5/x^; R^2^ = 0.989		9.37
2.76 M	y = e^ln(100)−2.7/(x−1.017)^; R^2^ = 0.896		4.96
5.52 M	y = e^ln(100)−4.0/(x−0.672)^; R^2^ = 0.989		6.43

## Data Availability

All data are available at Festetics Bioinnovation Group, Institute of Genetics and Biotechnology, Georgikon Campus, Hungarian University of Agriculture and Life Sciences.
